# Does electronic clinical microbiology results reporting influence medical decision making: a pre- and post-interview study of medical specialists

**DOI:** 10.1186/1472-6947-11-19

**Published:** 2011-03-30

**Authors:** Marjan J Bruins, Gijs JHM Ruijs, Maurice JHM Wolfhagen, Peter Bloembergen, Jos ECM Aarts

**Affiliations:** 1 Laboratory of Clinical Microbiology and Infectious Diseases, Isala klinieken, Stilobadstraat 3, 8021 AB Zwolle, The Netherlands; 2 Institute of Health Policy and Management, Erasmus University, PO Box 1738, 3000 DR Rotterdam, The Netherlands

## Abstract

**Background:**

Clinicians view the accuracy of test results and the turnaround time as the two most important service aspects of the clinical microbiology laboratory. Because of the time needed for the culturing of infectious agents, final hardcopy culture results will often be available too late to have a significant impact on early antimicrobial therapy decisions, vital in infectious disease management. The clinical microbiologist therefore reports to the clinician clinically relevant preliminary results at any moment during the diagnostic process, mostly by telephone. Telephone reporting is error prone, however. Electronic reporting of culture results instead of reporting on paper may shorten the turnaround time and may ensure correct communication of results. The purpose of this study was to assess the impact of the implementation of electronic reporting of final microbiology results on medical decision making.

**Methods:**

In a pre- and post-interview study using a semi-structured design we asked medical specialists in our hospital about their use and appreciation of clinical microbiology results reporting before and after the implementation of an electronic reporting system.

**Results:**

Electronic reporting was highly appreciated by all interviewed clinicians. Major advantages were reduction of hardcopy handling and the possibility to review results in relation to other patient data. Use and meaning of microbiology reports differ significantly between medical specialties. Most clinicians need preliminary results for therapy decisions quickly. Therefore, after the implementation of electronic reporting, telephone consultation between clinician and microbiologist remained the key means of communication.

**Conclusions:**

Overall, electronic reporting increased the workflow efficiency of the medical specialists, but did not have an impact on their decision-making.

## Background

Customer satisfaction has been reported as a critical performance measure for laboratory medicine, including clinical microbiology. Next to the accuracy of results physicians rated turnaround times (TAT) as the most important service aspect for clinical laboratories [1,2].

Diagnosing infectious agents is an essential component of clinical medicine. Requesting of clinical microbiology diagnostic tests by medical specialists is highly indication driven, which means that the clinician observes or suspects an infection and decides on laboratory tests to ascertain its exact nature. In situations that infections are likely to occur or pose a severe risk of patient morbidity or mortality, such as in intensive care medicine and neonatology, testing for infectious agents is often a guideline based, standard surveillance procedure.

When a bacterial infection is suspected that needs to be treated, antimicrobial therapy is started as soon as possible. The choice of antibiotics is usually based on the local antibiotic guidelines for the empirical treatment of infections. For an important part, clinical microbiology diagnostics involve culturing of patient samples for pathogenic micro-organisms. Prior to starting therapy a patient sample such as blood, urine or pus is collected and is sent to the microbiology laboratory together with a filled out request form. Technicians process the sample according to relevant laboratory algorithms, perform microscopy and culture, identify potential pathogens and establish antimicrobial susceptibilities. When all results are final and complete, the clinical microbiologist performs a final check and sends an authorized report to the requesting clinician. This final culture result report completes the microbiology request and is also of epidemiological importance.

Based on the often preliminary identification and susceptibility results of the infectious agent(s) adjustment of the treatment may be warranted to achieve higher efficacy, avoid development of antimicrobial resistance and to reduce costs [3]. The sooner appropriate therapy can be achieved, preferably within the first 24 hours, the better the patient's clinical outcome will be [4,5]. Because bacterial growth takes time, full culture results may only become available after two to seven days or even longer and may arrive too late to contribute to therapy decisions and may be of less interest to the treating physician. The patient may already have died, recovered or have left the hospital. Results are therefore often reported at intermediate stages. At any moment during the diagnostic process the clinical microbiologist will report new microscopy or preliminary culture results to the clinician by telephone or in person, either at the clinician's request or proactively, if results are deemed clinically relevant and needed to achieve appropriate therapy [6]. Often these messages include treatment advice. Vice versa the clinician will often consult the microbiologist to ask for preliminary first results if these are considered to be urgently needed for therapy decisions.

Telephone reporting therefore is a key means of communication in infectious disease management, however possibly more error prone than other means of communication because the information is not always correctly understood or handled by the recipient in the hospital.

Electronic reporting of clinical microbiology results instead of reporting on paper may help to shorten the often critical TAT and to ensure correct communication of results. In general, it might increase both the efficiency and the efficacy of medical practice and subsequently have a beneficial impact on patient outcome and healthcare costs [7].

In a pre- and post-interview study using a semi-structured design we asked clinicians about their use and appreciation of clinical microbiology results reporting before and after the implementation of an electronic reporting system, in order to assess its impact on medical decision making [8].

## Methods

### Setting

The study was conducted between December 2005 and June 2007 in the Isala klinieken, a multisite 1,100 bed, tertiary care teaching hospital in Zwolle, in the central-eastern part of The Netherlands. The medical facilities include general, cardiothoracic and neonatal intensive care units (ICU). The clinical microbiology laboratory serves the hospital, general practices and nursing homes in the area and processes approximately 110,000 samples per year.

Three clinical microbiologists are in charge of the laboratory. They are physicians (MDs) trained in clinical (or medical) microbiology, which is a recognized medical specialty now in most member states of the European Union [9]. They collaborate interdependently with other medical specialists, provide solicited and unsolicited advice on managing patients with infectious diseases and offer a 24 hour on-call service [6,10]. The frequency of consultation varies per medical specialty. Daily, the microbiologist visits the general ICU to discuss treatment strategies with the intensivist. Other specialists are telephoned to report microscopy or culture results deemed clinically relevant as soon as these have become available.

### Electronic reporting

The laboratory operates a laboratory information management system (LIMS). Microbiology results were communicated to the hospital on paper before 2006 and electronically after 2006, when the LIMS was connected to the hospital electronic medical record (EMR) system EriDanos. The EMR system EriDanos has been developed in-house and customized for use in the Isala klinieken since 2001. Besides patient records including medication and medical history, it contains results of diagnostic tests performed by the laboratories of clinical chemistry, pathology and microbiology, and all diagnostic imaging and results of other diagnostic procedures such as endoscopy. Microbiology requests are visible in EriDanos as pending as soon as the sample is registered into the LIMS. Final microbiology reports are transferred to the system once daily and can be viewed on screen by all authorized clinicians, dedicated nurses such as HIV nurse practitioners and general practitioners.

### Recruitment of subjects

Thirteen medical specialists, representing the specialties with the highest numbers of microbiology tests requests yearly, were recruited for this study (Table 1). The selected specialists were senior staff clinicians, who were responsible for the management of infectious disease patients on behalf of their specialty and who would be able to take part in both pre- and post-implementation interviewing. Thirteen physicians representing eleven specialties took part in pre-implementation interviews in December 2005 and twelve of them in post-implementation interviews in April - June 2007. Unfortunately no attending physician from obstetrics/gynaecology was available for interviewing. A second year resident was recruited instead, but could only be interviewed before implementation and was therefore excluded from the analysis.

**Table 1:** Recruited medical specialists

Specialty	Interviewed medical specialists		Number of microbiology culture requests (2005)
		
	Pre-implementation (2005)	Post-implementation (2007)	
General surgery	1	1	5,809
ICU medicine	2	2	4,210
Internal medicine*	3	3	11,780
Neonatology	2	2	3,636
Obstetrics/gynaecology	1	-	4,453
Orthopedic surgery	1	1	1,533
Paediatrics	1	1	2,995
Pulmonology	1	1	4,715
Urology	1	1	4,997

* Internal medicine included the medical specialties infectiology and oncology

### Interviews

Semi-structured interviews were held according to the schedule listed in Table 2 to assess how medical specialists valued result reporting before and after the implementation of an electronic reporting system. In the pre-implementation interviews the clinicians were asked about the use and management of test results in their practice. The post-implementation interviews addressed the use of electronic results reporting and ensuing changes in medical decision making. The pre-implementation interview lasted between 35 and 60 minutes and the post-implementation interview between 11 and 28 minutes. The interviews were recorded, transcribed and analysed using a manual theme coding method.

**Table 2:** Interviews

Interview schedule and topics	
**Pre-implementation**	**Post-implementation**

Description of medical and test requesting practice	
Use and meaning of test results reporting	Use and meaning of test results reporting
Problems and challenges	Impact of electronic results reporting
Expectations of electronic results reporting	Suggestions for improvement

Data validation was performed by iterative feedback with respondents, repeated immersing in data by the authors and triangulation. Triangulation was achieved by iterative discussion of the coded data and pre- and post implementation data comparison.

## Results

Table 3 shows the results of the two series of interviews, arranged by specialty.

**Table 3:** Results of interviewing medical specialists

	Pre-implementation			Post-implementation		
		
Medical specialty (no. of interviewees)	Description of requesting practice	Use and meaning of results reporting	Problems/Expectations	Results reporting; impact of electronic reporting	Suggestions for improvement	Other remarks
General surgery (1)	Indication based requesting. In OR sampling and clinical data by surgeon, in wards and ER by resident.	Final results are hardly looked at, have in 95% of cases no effect on treatment decisions. Phone calls by CL rare.	/Occasional, selective viewing.	Fast, selective viewing of results.		Epidemiological research in cooperation with CM would be useful.
ICU medicine (2)	Indication based requesting by clinician, protocol based requests by nurse.	First results come in from CM by telephone and at daily conference at ICU with CM present. Final hardcopy results less important.	Tracking of requests impossible. Often incomplete recording of oral consultation in medical record./Daily consulting must remain, also when results in EMR.	Still most information through daily meeting and telephone reporting by CM. No real changes in workflow. Complete and easier patient overview in EMR.	Preliminary results in EMR.	Close communication with CM must remain.
Infectiology (1)	Indication based requesting. Forms by clinician, sampling by nurse.	Therapy starts before results are available. Telephone reporting of relevant information by CM; phone calls for preliminary results mostly by residents. Hardcopies are always seen.	Occasionally results telephoned by CM misinterpreted by residents./Reports of every intermediate stage in culture workup.	Preliminary results still telephoned by CM, still many phone calls by CL. End to paperwork. Better overview. Review function important. No more double requests.	Electronic reporting of any interim result! Possibility to click away read results.	
Internal medicine (1)	Mostly indication based requesting. Sampling and forms by both clinician and nurse.	Telephone reporting of relevant information by CM; CL calls if necessary.	Delay in hardcopy delivery. Often hardcopy reports lay unattended for some time./Preliminary results (first growth) in EMR: fewer phone calls necessary.	Fewer phone calls by CL. Faster workflow. Easier access; better overview. Request marked as pending is appreciated. System commands disciplined viewing.	Possibility to perform epidemiology. Implementation of complete electronic communication with GPs.	
Neonatology (2)	Both protocol and indication based requesting. Sampling and forms by both clinician and nurse.	Telephone reporting of relevant information by CM; many phone calls by CL for preliminary results. Stat request if necessary. Final results mainly used for follow-up and epidemiology.	TATs and logistics for sending results; delay in hardcopy delivery./Overview in EMR. Retrospective analysis of data. Possibility to perform epidemiology.	Still many phone calls by CL. Better overview of patient data. Patient information easier to find and retrieve.	Shorter TATs. Preliminary results in system. Usability of EMR system: many pages; layout. Trending is still difficult. Cumulative overview per patient. Bedside access of patient data.	PDMS on wish list. Age related background information with final results would be useful.

	**Pre-implementation**			**Post-implementation**		
		
**Medical specialty** **(no. of interviewees)**	**Description of requesting practice**	**Use and meaning of results reporting**	**Problems/Expectations**	**Results reporting; impact of electronic reporting**	**Suggestions for improvement**	**Other remarks**

Oncology (1)	Both protocol and indication based requesting. Sampling and forms by both clinician and nurse.	Telephone reporting of relevant information by CM; CL calls if necessary. Once weekly conference with CM present. Final results rarely affect treatment. Hardcopy results that have no consequences usually thrown out. TAT's no problem.	Tracking of requests impossible. Logistics of keeping paper written results./Reduction of paper. Historic overview of results in EMR. Possibility to perform epidemiology.	Relevant preliminary results still telephoned by CM, phone call by CL quicker than electronic report. Disciplining of reviewing data. Better reviewing. No more lost reports. Integral overview of patient. Viewing possible at any workstation.	Usability: EMR system is slow; layout of results. Important results should be marked.	
Orthopaedic surgery (1)	Indication based requesting. Sampling by surgeon, forms by nurse except in OR.	Hardcopy reports arrive in mailbox. Therapy started when specimen was taken. In case of urgency phone call by CL.	Delay in hardcopy delivery; problems with reporting over weekends./Integral overview of all diagnostic testing including clinical microbiology.	Quicker availability of results, so fewer calls by CL for final results. Still need for preliminary results. Better reviewing and overview. Administrative time saved. Request marked as pending is appreciated.	Usability.	Regular meeting on complications with CM present.
Paediatrics (1)	Indication based requesting by clinician. Sampling mostly by nurse.	Telephone reporting of relevant information by CM. Therapy only changes if result contradicts expectation. Once weekly conference with CM present.	Logistics: large number of hardcopy results. All reports are seen by paediatricians though, chance of missing results small. Availability of results takes too long./Easy access to results in EMR. Better overview. Historical results easier to retrieve.	Fewer phone calls by CL. Availability of results and patient data at large good. Better logistics and faster reporting. No more lost reports. Administrative time saved.	Cumulative overview per patient.	
Pulmonology (1)	Indication based requesting. Sampling by resident or nurse, because sometimes difficult to time.	Clinical insight (experience) takes precedence over results. Usually no need for fast reporting. Phone call or stat request (Gram stain) in case of urgency by CL.	Hardcopy filing and locating. Results sometimes remain unseen./Better retrospective analysis. Better logistics. Possibility of susceptibility trend analysis.	System not used frequently. Convenient reviewing, convenient overview, even helps prevent errors.	No trending. Important results should be marked.	
Urology (1)	Indication based requesting. Sampling by patient, chemistry lab or nurse.	Preliminary results important to start treatment. Phone calls by CL. Hardcopies are always seen.	Difficult to get integrated picture. Delay in hardcopy delivery./Integral overview of patient information including results of requests by GP or other specialty.	Phone calls by CL only for therapy advice. Daily viewing of results. Request marked as pending appreciated; no more double requests. No more lost reports. Integral overview of patient. Viewing possible at any workstation.		

CL, clinician; CM, clinical microbiologist; ER, emergency room; PDMS, data management system explicitly developed for use in paediatric and neonatal intensive care units.

### Description of test requesting practice

Most microbiology requests are indication based to confirm and analyze an infectious disease. For certain vulnerable patient groups (ICU, neonatology) samples for culture are also collected for epidemiological purposes. The treating medical specialist is responsible for diagnostic requests but often delegates the administrative work and the specimen collection to residents and nurses.

### Pre-implementation

The use and meaning of test results varied significantly between the different medical specialties. The clinicians in the ICU relied on the daily contacts with the clinical microbiologist to obtain the latest results on which to base the treatment course, and not on hardcopy reports. In general surgery hardcopy final results were hardly used and even thrown away. Most other clinicians said they read reports daily, but complained about delays in receipt, and the handling time and errors involved with filing of the numerous papers. Additional to the telephone reporting of new relevant results by the clinical microbiologist, clinicians or residents of all specialties frequently phoned the laboratory to ask whether requested tests were indeed being processed or to obtain preliminary results, because waiting for the final results would take too long. Other general problems mentioned were the lack of overview of a patient's history, and the difficulties in doing epidemiological research.

### Post-implementation

All interviewed clinicians highly appreciated the availability of results reporting in the EMR system. The advantages reported by the interviewees included easier and faster access to microbiology reports, reduction of hardcopy handling, a better and complete overview of patient data and history because of the possibility to review microbiology results in relation to other patient data, better possibilities for clinical research and the fact that a request received for processing was marked in the system. The easy access to the EMR system at any network workstation was appreciated, however, the neonatologists preferred to have the paper medical chart accessible at the bedside.

According to the interviewees, request patterns and organization had not changed after the implementation of electronic reporting. Most clinicians did not change their daily report viewing routine after the implementation.

Telephoning the laboratory to ask if a specimen had indeed arrived was no longer necessary, because every received request was promptly marked as such in the EMR. Along with the loss of hardcopy filing and searching, this saved substantial time in the medical practice and improved the efficiency. However, electronic reporting did not change the process of decision making, because the necessary early information was not shown in the EMR and was only available by telephone. The interviewed specialists reported that the number of phone calls they had to make to obtain preliminary first results did not decrease after implementation. The ability to view first preliminary results in the EMR was the main suggestion for improvement.

The interviews clearly showed how much the use and meaning of microbiology reports differ between medical specialties and respective patient types. At the onset of infection, empirical treatment is started that may need to be adjusted based on the culture results. Since culturing and its complete work-up takes at least two days, final results will often come in too late to make a difference. According to our study, the requesting clinicians might roughly be divided into three microbiology report user groups. For the closely monitored ICU-patients first results such as microscopy and first bacterial growth are very important. Reporting will continue to be fastest by telephone and during the daily conferences at the ICU at which the clinical microbiologist takes part. Electronic reporting was considered useful for reviewing patients, but obviously brought no real changes in the ICU-clinicians' workflow or decision making.

A second group consists of medical specialists (general surgery, pulmonology) who indicated that for their patients final culture results will only in rare cases lead to a change of the empirical antimicrobial treatment that was initially started based on hospital antibiotic guidelines or experience. The patient may already have been discharged before the final report becomes available, may have died or is improving despite apparently inappropriate treatment. Final results are therefore hardly looked at. Here first results are almost never needed, but can still be obtained by telephone in case of an emergency while a safety net is provided by the clinical microbiologist who will always telephone new interim information deemed clinically relevant.

The third, most interesting user group is the majority of clinicians who want to use preliminary information such as microscopy results for treatment decisions and who expected those to be reported electronically. In implementing the EMR microbiology module a deliberate choice was made to not show preliminary results. The clinical microbiologists prefer peer consultation by telephone, because it is the fastest way to reach the right person, it ascertains that the message has been conveyed correctly and it gives the opportunity to discuss diagnosis, treatment and any necessary infection control measures. Another consideration has been that preliminary results may change when the definitive results are ready, which may lead to misunderstanding and error. A review paper on quality assurance of clinical microbiology test results argues that information developed as part of the diagnostic process is not easily amenable for unambiguous reporting [11].

As a result, this third group of clinicians reported no reduction in phone calls concerning preliminary results, which was corroborated by the clinical microbiologists. This is an important finding, which indicates that for the largest microbiology report user group therapy decision making remains to be dependent on telephone reporting and is not affected by electronic reporting.

### Limitations of the study

Interviews were limited to one and if possible two representatives of the medical specialties with the highest number of microbiology test requests yearly. The senior clinician in whose name the most requests were made was asked to participate in the study. In daily practice however, it is often residents, junior doctors and nurses who actually take care of the request forms and sampling. Moreover, in some departments for the sake of convenience and because there is no system of individual request registration all specialists use this one colleague's name for requesting tests. Interviewing the same persons before and after implementation, enabled us to get a true impression of the experienced changes resulting from electronic reporting. The findings of the study may be biased because they are based on interviews that reflect the specialists' views and perceptions. In order to reduce bias the interviews were conducted by an independent university based researcher using a strict interview items protocol (Table 2).

## Discussion

Published research on the impact of electronic reporting of clinical microbiology results on medical decision making in the hospital is disappointingly scarce, especially considering the vast amount of resources devoted to hospital EMR systems [12]. The positive effects on the clinicians' and nurses' workflow found in our study concur with the few available reports on the benefits of electronic reporting experienced by physicians [13,14]. Despite this increased efficiency in medical practice however, earlier studies do not indicate improvements in the clinical efficacy in terms of morbidity and mortality. In our previous study, where the diagnostic process after the first culture step was accelerated by using an automated system for bacterial identification and susceptibility testing and same day hardcopy reporting, no beneficial clinical impact was measurable [15]. A similar Dutch study showed a reduction in antibiotic use but not in mortality [16]. Our findings in the current study indicate that also after implementing electronic reporting critical therapy decisions will still be based on empirical guidelines and telephoned first results and least on final culture reports [17,18].

In the interviews clinicians reported three problems of telephone communication. The clinician who requested the test may not be reached at the phone, the message may not be completely understood by the person answering the call, or a written note containing information about the call may not reach the clinician in time. In a study of telephone reporting Barenfanger *et al*. suggested that it should adhere to a guideline including repeating the information that was communicated [19].

Another solution to this problem and a wish come true for many clinicians, might be that preliminary test results become part of electronic results reporting even though the final authorized result may differ. The preliminary test result should be seen as the best state of knowledge that a clinician needs to decide on a therapy. Each new entry should be provided with a recommendation how to interpret and use the information [20]. As such, a change could not be construed as correcting an error and the preliminary status would be emphasised to caution the requesting physician.

Still, in certain cases telephone reporting of critical first results including clinical advice and clarification if needed will be preferable. Moreover, studies show that personal consultation between clinical microbiologist and clinician ensures the highest efficacy in infectious disease patient management [6,21].

## Conclusions

Medical specialists value electronic reporting of clinical microbiology, because it increases the efficiency in their medical practice and saves valuable time. Final culture results may be available sooner compared to the former reporting on paper, but, in contrast to current opinions, this shorter turnaround time does not automatically influence medical decision making. Where the fast reporting of first results is of importance, telephone reporting is still the communication method of choice. The conveying and recording of telephone messages may need improvements and future research should determine how electronic reporting of early, preliminary culture results may be implemented to be of additional use.

## Competing interests

The authors declare that they have no competing interests.

## Authors' contributions

MB participated in the conception and design of the study, and in the analysis and interpretation of the data and drafted and revised the manuscript. JA participated in the conception and design of the study, conducted the interviews, participated in the analysis and interpretation of data, and in the writing and revising of the manuscript. GR was involved in the conception of the study, interpretation of the data, and revising the manuscript critically. MW and PB were involved in revising the manuscript.

All authors read and approved the final manuscript.

## Pre-publication history

The pre-publication history for this paper can be accessed here:

http://www.biomedcentral.com/1472-6947/11/19/prepub
